# Potential Role of eNOS Genetic Variants in Ischemic Heart Disease Susceptibility and Clinical Presentation

**DOI:** 10.3390/jcdd8090116

**Published:** 2021-09-18

**Authors:** Paolo Severino, Andrea D’Amato, Silvia Prosperi, Michele Magnocavallo, Marco Valerio Mariani, Lucrezia Netti, Lucia Ilaria Birtolo, Paolo De Orchi, Cristina Chimenti, Viviana Maestrini, Fabio Miraldi, Carlo Lavalle, Viviana Caputo, Raffaele Palmirotta, Massimo Mancone, Francesco Fedele

**Affiliations:** 1 Department of Clinical, Internal, Anesthesiology and Cardiovascular Sciences, Sapienza University of Rome, Viale del Policlinico 155, 00161 Rome, Italy; damatoandrea92@gmail.com (A.D.); silviapro@outlook.it (S.P.); michelefg91@gmail.com (M.M.); marcoval.mariani@gmail.com (M.V.M.); lucrezia.netti@gmail.com (L.N.); ilariabirtolo@gmail.com (L.I.B.); paolo.deorchi95@gmail.com (P.D.O.); cristina.chimenti@uniroma1.it (C.C.); viviana.maestrini@uniroma1.it (V.M.); fabio.miraldi@uniroma1.it (F.M.); carlo.lavalle@uniroma1.it (C.L.); massimo.mancone@uniroma1.it (M.M.); francesco.fedele@uniroma1.it (F.F.); 2 Department of Experimental Medicine, Sapienza University of Rome, Policlinico Umberto I Hospital, Viale Regina Elena 324, 00161 Rome, Italy; viviana.caputo@uniroma1.it; 3 Interdisciplinary Department of Medicine, School of Medicine, University of Bari ‘Aldo Moro’, 70124 Bari, Italy; raffaele.palmirotta@uniba.it

**Keywords:** ischemic heart disease, endothelial dysfunction, endothelial nitric oxide synthase, single-nucleotide polymorphism, acute coronary syndrome

## Abstract

**Background:** IHD is determined by an inadequate coronary blood supply to the myocardium, and endothelial dysfunction may represent one of the main pathophysiological mechanisms involved. Genetic predisposition to endothelial dysfunction has been associated with IHD and its clinical manifestation. However, studies are often confounding and inconclusive for several reasons, such as interethnic differences. Validation of results in larger cohorts and new populations is needed. The aim of this study is to evaluate the associations between the allelic variants of the eNOS rs1799983 single-nucleotide polymorphism, IHD susceptibility and its clinical presentation. **Methods:** A total of 362 consecutive patients with suspected myocardial ischemia were enrolled. Patients were divided into three groups: G1, coronary artery disease (CAD); G2, coronary microvascular dysfunction (CMD); and G3, a control group with anatomically and functionally normal coronary arteries. Analysis of three allelic variants, GT, GG and TT, of rs1799983 for the NOS3 gene, encoding for eNOS, was performed. **Results:** rs1799983_GT was significantly more expressed by the ischemic groups (G1 and G2) compared to G3. The TT variant was significantly more expressed by the G1 group, compared to the G2 group. Among ischemic patients, GT was significantly more expressed in patients with acute coronary syndrome (ACS) presentation, compared to other clinical presentations. In the multivariate analysis, the allelic variant GT was found to potentially represent an independent predictor of IHD and ACS presentation. **Conclusion:** The presence of the SNP rs1799983_GT, encoding for eNOS, is an independent risk factor for IHD and, remarkably, for ACS presentation, independently of cardiovascular risk factors. These results may be useful for the prediction of IHD development, particularly with an acute clinical manifestation. They may allow the early identification of patients at high risk of developing IHD with an ACS, promoting a genetic-based prevention strategy against IHD.

## 1. Introduction

In the pathophysiology of ischemic heart disease (IHD), genetic susceptibility is often ignored or not sufficiently investigated. The atherosclerotic involvement of large, epicardial coronary arteries, which leads to vessel obstruction, is often mentioned as synonymous with IHD [1]. Coronary artery disease (CAD) is a pathological process characterized by atherosclerotic plaque formation and progression, which may cause various degrees of myocardial ischemia, reducing coronary blood flow to the myocardium [2]. CAD may be associated with different clinical presentations of IHD, from acute coronary syndrome (ACS), when an acute plaque destabilization occurs, to chronic coronary syndrome (CCS), when plaque growth occurs progressively and slowly [2]. CAD is the leading cause of myocardial ischemia, but not the only one. In fact, coronary artery obstructive lesions have not been found in up to 70% of patients with angina and confirmed myocardial ischemia, at coronary angiography [2,3,4]. Ischemia with non-obstructive coronary arteries (INOCA) is determined by a mismatch between the myocardial metabolic demand and coronary arteries’ blood supply, in the absence of significant coronary obstruction [1]. Myocardial infarction with non-obstructive coronary arteries (MINOCA) is a clinical condition defined by criteria relating to the presence of acute myocardial infarction, in accordance with the Fourth Universal Definition of Myocardial Infarction [5], in the absence of obstructive atherosclerotic lesions involving >50% of large epicardial vessels’ diameter, and with an overt, clinical, specific cause responsible for the acute presentation [1,6,7]. In this regard, coronary microvascular dysfunction (CMD) may represent one of the main pathophysiological mechanisms involved in INOCA and MINOCA [1,3,4,7,8].

CMD has been observed as a pathophysiological mechanism in up to 20% of acute coronary syndromes (ACSs) and about 50% of chronic coronary syndromes (CCSs) [1]. The coronary microvascular compartment, made up of arterioles and capillaries with a diameter of <500 µm, is the main regulator of total coronary resistances [8,9,10,11,12,13,14]. CMD is determined by functional arteriolar disorders and microvascular structure remodeling [3,15]. Although CV risk factors and atherosclerosis mainly involve larger epicardial arteries, the coronary microcirculation should also be a target. However, CMD promotes atherosclerotic disease development and progression, at the epicardial arteries, through continuous blood slowdown [1,3,7,8,9,10,11,12,13,14].

**Figure 1:**
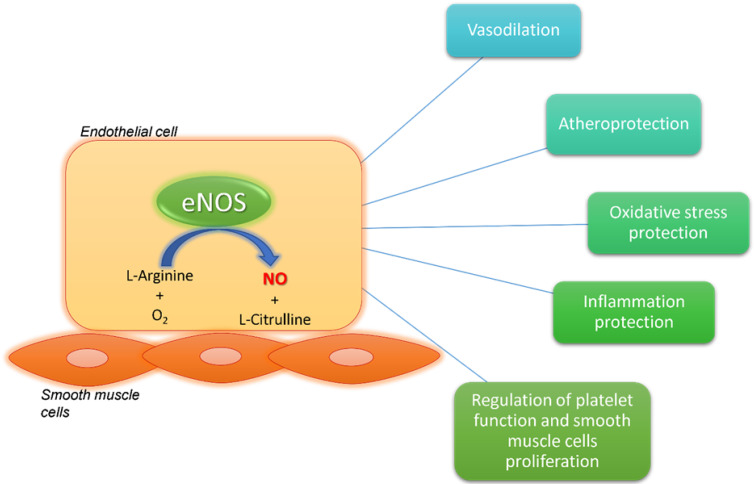
Endothelial nitric oxide synthase (eNOS) and nitric oxide (NO) physiological effects. eNOS produces NO starting from L-arginine and oxygen. NO has several beneficial effects on the vasculature. It is involved in atheroprotection and vasodilation. It contrasts oxidative stress, inflammation, platelet dysfunction and smooth muscle cell proliferation. eNOS: endothelial nitric oxide synthase; NO: nitric oxide; O_2_: oxygen.

For this reason, myocardial ischemia is more complex than a simplistic process attributable to a single pathophysiological process involving a single coronary district. The coronary microcirculation and larger epicardial arteries should not be considered as two independent compartments. They represent an interconnected network, continuously in communication, sharing the same basic pathophysiological mechanisms that lead to myocardial ischemia. In this context, endothelial dysfunction is one of the most relevant causes contributing to myocardial ischemia [16]. It promotes CMD, but also contributes to coronary atherosclerosis. The endothelium is the inner layer of vessels’ walls, and it has a main role in vascular homeostasis and microvascular function [16,17]. In terms of its physiological condition, the endothelial function is guaranteed by the production of vasoactive molecules, particularly nitric oxide (NO), which plays an atheroprotective and vasodilatory role (Figure 1). NO is produced by endothelial nitric oxide synthase (eNOS) starting from oxygen and L-arginine [16,17].

Although the role of CV risk factors is well established, just recently, a role in genetic susceptibility for endothelial dysfunction has been taken into consideration, with regard to IHD predisposition. In this context, genetic susceptibility may play a role comparable to that of traditional CV risk factors. In particular, some single-nucleotide polymorphisms (SNPs) of the eNOS-encoding gene may be involved in myocardial ischemia susceptibility, through endothelial dysfunction [16]. In this context, several studies have demonstrated the associations between eNOS genetic variants, IHD susceptibility and its clinical presentation [18,19]. However, they are often inconclusive or conflicting, and they do not reach significance in terms of disease prediction [18,19,20]. Most of the results obtained from these studies are circumscribed to specific ethnicities and populations. In fact, ethnicity is responsible for heterogeneity and controversies, both in terms of genotype background and gene–environment interaction [20]. The interactions between the eNOS gene and other genes, as well as gene–environment interactions, may have an impact on the polymorphism function and, therefore, on pathophysiological consequences and clinical presentation [20]. For this reason, the reproducibility of results in larger cohorts and with other ethnic groups is a primary target for the validation of the genetic predictors of IHD and ACS, which may be helpful for prognostic stratification, disease risk prediction and therapeutic management.

In this context, we previously described that the single-nucleotide polymorphism (SNP) rs1799983 (G894T; Glu298Asp) in exon 7 of the NOS3 gene on chromosome 7q35-36, encoding for eNOS production, with a key role in vascular homeostasis, vasodilation and endothelial function preservation [16,17], is associated with endothelial dysfunction and susceptibility to IHD [21].

The aim of this study is to evaluate the association among allelic variants of the NOS3 rs1799983 SNP, IHD susceptibility and its clinical presentation.

## 2. Materials and Methods

In this observational, single-center, prospective study, 362 consecutive patients admitted to the Department of Clinical, Internal, Anesthesiology and Cardiovascular Sciences of Sapienza University of Rome, due to IHD, and with an indication for coronary angiography, were enrolled between February 2019 and February 2021. A coronary angiography was performed according to current guidelines [22].

Inclusion criteria were as follows:

(1) Documented or suspected acute or chronic coronary syndrome with an indication to perform coronary angiography according to current guidelines [22];
(2) Age >18 years;
(3) Written informed consent.

Exclusion criteria were as follows:

(1) Cardiogenic shock;
(2) Other known genetic diseases.

The following baseline features were recorded in a dedicated database: admission and discharge dates, previous medical history, gender, age, CV risk factors and medical therapy. All patients signed a consent form, and the present study was approved by the Policlinico Umberto I Ethical Committee. All procedures were executed following the 1975 Helsinki Declaration and the ethical standards of the institutional and national research committees.

### 2.1. Study Protocol

The study protocol provided a comprehensive cardiological evaluation including a 12-lead electrocardiogram (ECG), physical examination, echocardiogram before and after coronary invasive evaluation, peripheral blood sample collection to evaluate blood count and patients’ metabolic status. Informed consent was obtained from all subjects involved in the study. In all enrolled patients, an adjunctive peripheral blood sample was taken to perform genetic analysis.

All enrolled patients underwent coronary angiography, performed through the insertion of a sheath in the radial artery access, using the Judkins approach. Patients with significant CAD were treated according to current guidelines [22]; patients with absence of coronary artery obstructive lesions underwent functional intracoronary tests to evaluate the presence of CMD. A Doppler flow wire was used to evaluate endothelial microvascular function, through intracoronary 2.5–10 μg acetylcholine infusion [23]. The coronary microvascular function was estimated through coronary flow reserve (CFR) evaluation.

According to the results of the coronary angiography evaluation and intracoronary functional tests, all enrolled patients were divided into three groups:

G1: patients affected by CAD, defined by the presence of an atherosclerotic plaque, which determines a ≥50% coronary stenosis;

G2: patients with CMD, defined by a CFR ≤2.5 after intracoronary acetylcholine infusion, and with anatomically normal coronary arteries, evaluated through coronary angiography;

G3: patients with anatomically and functionally (CFR ≥2.5 after intracoronary acetylcholine infusion) normal coronary arteries.

G1 and G2 were characterized by patients with diagnosed myocardial ischemia, while G3 represented the control group, made up of normal, healthy patients.

### 2.2. Genetic Analysis and Polymerase Chain Reaction

According to international guidelines and the study protocol, anticoagulated EDTA whole blood samples were collected from each patient and cryopreserved at −80 °C until subsequent DNA analysis [24].

Genomic DNA was isolated using the ISOLATE II genomic DNA kit (Bioline Reagents Ltd., Meridian Bioscience, London, UK), an ionic exchange column-based kit, according to the manufacturer’s instructions. The rs1799983 polymorphism was determined by a standard polymerase chain reaction (PCR) amplification using the HotStarTaq Master Mix kit (QIAGEN Inc., Valencia, CA, USA) in a GeneAmp PCR System 9700 (Life Technologies, Carlsbad, CA, USA) as follows: a first step of DNA denaturation at 95 °C for 15 min, 32 cycles at 94 °C for 30 s, 58 °C for 30 s and 72 °C for 1 min, and a final extension step at 72 °C for 10 min. Primers (F5′-CATGAGGCTCAGCCCCAGAAC-3′ and R5′-AGTCAATCCCTTTGGTGCTCAC-3′) were selected from the NOS3 Ensemble sequence database (Ensembl:ENSG00000164867) and designed using the free web-based application Primer3Plus (https://www.bioinformatics.nl/cgi-bin/primer3plus/primer3plus.cgi (accessed on 10 September 2021). Direct sequencing analyses were performed in both forward and reverse strands with the same pair of primers using the Big Dye Terminator v3.1 Cycle Sequencing kit (Applied Biosystem, Waltham, MA, USA) and run on a 3500 Genetic Analyzer (Thermo Fisher Scientific Inc., Waltham, MA, USA).

### 2.3. Cardiovascular Risk Factor Definitions

Familial history of CV disease was defined by the presence of at least one first-degree relative who experienced a cardio- and/or cerebrovascular accident, before the age of 60. Arterial hypertension was defined by anti-hypertensive therapy assumption or presence of a systolic and diastolic blood pressure higher than 140 mmHg and 90 mmHg, respectively, in at least three measurements. Diabetes mellitus was defined by anti-diabetic drug assumption or fasting glucose values >126 mg/dL, in at least three measurements. Hypercholesterolemia was defined by cholesterol-lowering drug assumption or total cholesterol >200 mg/dL. Patients were defined as having a smoking habit if they smoked tobacco or if they suspended tobacco smoking for less than 12 months, at the enrolment time.

### 2.4. Statistical Analysis

The sample size could be calculated assuming a 15% prevalence of normal macrovascular and microvascular coronary findings in unselected patients undergoing coronary angiography. We estimated that a sample size of at least 150 patients could enable the computation of two-sided 95% confidence intervals for such prevalence estimates ranging between −5.0 and +5.0%. Normal distribution of variables was assessed with the Kolmogorov-Smirnov test. For continuous variables, descriptive statistics were used (number of available observations, mean, median, standard deviation), while the median (interquartile range) was used for non-normal data. Categorical data were described as a number (percentage). Baseline demographic and clinical characteristics will be presented in table format. Student’s *t*-test, the χ2 test and the Fisher exact test were used for comparisons. Differences between variables with a non-normal distribution were tested with the Mann–Whitney *U* test. For all tests, a *p*-value less than 0.05 was considered statistically significant. The observed numbers of each genotype were compared with those expected for a population in the Hardy–Weinberg equilibrium using a free web-based application.

The odds ratios (ORs) and their 95% confidence intervals (95%CIs) were calculated to estimate the association between genetic polymorphisms and IHD by logistic regression analysis. Univariate and multivariate analyses were performed, and all the variables with a significant association (*p*-value < 0.10) in the univariate analysis were included in the multivariate analysis. The statistical analysis was performed using SPSS version 25.0 for Windows (IBM Software, Inc., Armonk, NY, USA).

## 3. Results

A total of 362 consecutive patients were enrolled in this study with an indication to undergo coronary angiography, due to suspected myocardial ischemia. A total of 218 patients were included in G1, 54 in G2 and 90 in G3. A total of 67% (245) of the total population was represented by the male gender, and the mean age was 64 ± 13 [median 66 (IQR: 56–74)]. The mean left ventricular ejection fraction (LVEF) at admission was 49 ± 11% [median 52 (IQR: 45–55)].

The baseline features of the population and the differences between the control group (G3) and ischemic groups (G1 and G2) are listed in Table 1.

Statistically significant differences were found among the three groups regarding CV risk factor prevalence and LVEF. In particular, arterial hypertension (*p* < 0.001), diabetes mellitus (*p* = 0.01), dyslipidemia (*p* = 0.03) and familial history of CV diseases (*p* = 0.001) were prevalent in the ischemic groups (G1 and G2), compared with the control group (G3), and LVEF was higher in normal patients compared with the ischemic groups (*p* < 0.001).

The baseline features and differences between the G1 and G2 patients are listed in Table 2.

Significant differences were observed between the G1 and G2 groups regarding LVEF and CV risk factor prevalence. Arterial hypertension (*p* = 0.001), diabetes mellitus (*p* = 0.01), dyslipidemia (*p* = 0.04) and smoking habit (*p* = 0.01) prevailed in G1. G1 showed a lower LVEF compared with G2, at admission (*p* = <0.001).

The allelic variant GT of the *NOS3* SNP rs1799983 was statistically significantly expressed by the ischemic groups (G1 and G2) compared with the control group G3 (OR: 2.36; CI 95% 1.08–5.2; *p*: 0.03). No differences were observed regarding the GT allelic variant of rs1799983 between the G1 and G2 groups (*p* = 0.47), while the TT allelic variant of rs1799983 was significantly expressed by the G1 group, compared with the G2 group (*p* < 0.001). In the multivariate analysis, the allelic variant GT of the SNP rs1799983 may represent an independent predictor of IHD (OR: 3.87; CI 95% 1.55–9.64; *p*: 0.004) (Table 3).

Subsequently, we observed an association among the three allelic variants, GG, GT and TT, of the SNP rs1799983 and myocardial ischemia clinical presentation. In the univariate analysis, we observed an association between the GT allelic variant and ACS presentation, at hospital admission, compared with patients without ACS presentation (OR 2.26; CI 95% 1.28–4; *p*: 0.005). In the multivariate analysis, the GT allelic variant may represent an independent predictor of ACS presentation (OR: 2.82; CI 95% 1.42–5.59; *p*: 0.003) (Table 4).

## 4. Discussion

The present study focused on the association between eNOS genetic variants and IHD susceptibility, with particular regard to myocardial ischemia clinical presentation. IHD, and particularly ACS, is an important mechanism leading to a high mortality and morbidity burden, as well as elevated healthcare costs, predisposing to both early and long-term complications, such as arrhythmic events and heart failure [6,25]. Most of the time, IHD is associated with the presence of traditional CV risk factors and atherosclerotic disease involving larger epicardial arteries. Just recently, a determinant role of genetic susceptibility has been taken into consideration. In this context, genetic susceptibility may play a role comparable to that of traditional CV risk factors, in terms of IHD susceptibility [26]. From the pathophysiological point of view, IHD is classically associated with atherosclerosis and CAD. However, patients with CAD do not always develop myocardial ischemia, and patients with an ischemic event do not always show an overt CAD. In this context, other ischemic pathophysiological mechanisms, such as CMD, should be considered. In fact, the coronary microcirculation represents the main site of coronary resistance regulation, while larger coronary arteries mainly have a conductance role [8,9,10,11,12,13,14]. However, although they have different roles, the coronary microcirculation and larger epicardial arteries should be not considered as two independent watertight compartments. They represent an interconnected network, continuously in communication, through several mechanisms and molecules.

In this regard, NO and endothelial cell function are important for coronary vascular homeostasis, to guarantee an adequate blood flow to the myocardium, according to the cardiomyocyte metabolic demand [16]. NO has several atheroprotective effects contrasting oxidative stress, inflammation, platelet dysfunction and smooth muscle cell proliferation. Endothelial dysfunction includes a wide spectrum of pathological phenotypes associated with several vascular alterations, such as vascular tone dysregulation, vessel wall inflammation and hyperpermeability, as well as atherosclerotic lesion progression [16] (Figure 1). For this reason, it is understandable how endothelial dysfunction may contribute to myocardial ischemia, affecting each vascular district at the coronary level, from the epicardial arteries to the microcirculation [1,7,16].

In this context, we believe that the study of genetically induced endothelial dysfunction may represent an important aspect for the comprehension of myocardial ischemia pathophysiology and clinics. In fact, several studies related eNOS SNPs with IHD and its clinical presentation, although they were often unable to predict the risk of developing myocardial ischemia, in a prospective setting [18,19]. Ciftçi et al. found that the eNOS -786C/C genotype was significantly represented in ACS patients, compared to the control group and patients with CAD, while -786TT was associated with CAD [27]. These results emphasize that eNOS plays a role in ACS pathogenesis, participating in the different pathophysiological mechanisms, leading to myocardial infarction [27]. The G894T SNP of eNOS is associated with ACS, also when corrected for the traditional CV risk factors [20,28]. Kukava et al. analyzed the association of different gene variants, comparing a group with myocardial infarction to a control group. They found a significant association between myocardial infarction and lipid metabolism-related gene variants, such as lipoprotein lipase (LPL), apolipoprotein E (APOE) and proprotein convertase subtilisin/kexin type 9 (PCSK9), as well as eNOS gene variants [18]. Regarding the eNOS gene variants, the polymorphism rs2070744 of eNOS, located in the promoter region, was associated with myocardial infarction [29]. Three eNOS gene allelic variants, -786TC, -922AG and -1468TA, in the 5′-flanking region have been found to impact the pathogenetic mechanisms of myocardial infarction [30]. Moreover, when these variants were associated with cigarette smoking, the risk of myocardial infarction increased, probably due to an interaction between the gene and environment [30]. Recently, Junctional cadherin 5 associated (JCAD) locus was identified as a risk factor for CAD and myocardial infarction, through endothelial dysfunction [31].

However, results regarding the association between eNOS genetic variants and IHD susceptibility and clinical presentation are often conflicting or inconclusive [20]. In this context, Pawlik et al. demonstrated that there is no association among the *NOS3* rs1799983 and rs2070744 polymorphisms and unstable angina [32]. Additionally, Vargan-Alarcon et al. found no differences regarding the eNOS polymorphism distribution between patients with ACS and healthy control groups [33]. Although Jo et al. found that the -786TC polymorphism had a frequent association with myocardial infarction in the Korean population [7], the eNOS4a allele, linked to the same polymorphism, showed a protective effect for ACS in the Korean population [34], while Hibi et al. found no association between the same polymorphism and myocardial infarction [35]. Isordia-Salas et al. showed that the eNOS polymorphism G894T (Glu298Asp) represents an independent risk factor for early STEMI presentation in the Mexican population, under 45 years [36], underlying the importance of genetic predisposition to endothelial dysfunction, considering the young age of people included in the study.

One of the main limits of these studies regards the aspect that most of these results were often circumscribed to specific ethnicities and populations. In fact, ethnicity is responsible for association heterogeneity and controversies, both in terms of genotype background and gene–environment interaction. The interaction among the eNOS gene and other genes, as well as gene–environment interaction, may have an impact on polymorphism function [18]. In this context, several studies have shown an association between the eNOS rs1799983 polymorphism and IHD in different populations, although in some cases, this association has not been demonstrated, probably due to interethnic differences [32]. For this reason, significant results on larger and new cohorts of patients are needed to validate new genetic predictors of IHD and ACS.

Given the recent findings and starting from our previous results on the protective role of coronary ATP-sensitive potassium channel genetic variants against IHD [21,37], we studied the possible association among allelic variants of the rs1799983 *NOS3* SNP, IHD susceptibility and its clinical presentation. Our data demonstrate that the SNP rs1799983_GT for *NOS3* was prevalent in ischemic patients with both CAD and CMD (G1 and G2 groups). Moreover, the same allelic variant was prevalent in patients with documented myocardial ischemia (G1 and G2 groups) and ACS presentation, at hospital admission, independently of CV risk factors. These associations were confirmed through multivariate analysis, which demonstrated that the allelic variant GT of the rs1799983 *NOS3* SNP is an independent predictor of IHD and ACS presentation. Moreover, according to the Hardy–Weinberg principle, there is a disequilibrium regarding the allelic distribution of the *NOS3* gene. This aspect strengthens the hypothesis that the SNP rs1799983_GT is an IHD predictor. We believe that the SNP rs1799983_GT for *NOS3* is associated with a dysregulation of eNOS, which causes an imbalance in NO activity.

The rs1799983 polymorphism in *NOS3* exon 7 determines the substitution of a guanine (G) with a thymine (T) at position 894 and a consequent modification of the coding sequence with an aminoacidic change from glutamic acid to aspartic acid, at position 298. Previous studies have shown that the presence of the T allele is associated with lower mRNA levels, leading to a reduction in eNOS expression and resulting in reduced vasoprotective action and endothelial damage [38,39]. However, a more recent in silico study showed that the Glu298Asp polymorphism, located on the surface of the protein, does not result in a structural change that could justify an impairment of function. However, the amino acid substitution may affect the interaction of the eNOS oxygenase domain with its several functional partners, such as Caveolin-1 (CAV1), Heat shock protein 90 alpha family class A member 1 (HSP90AA1), Calmodulin 1 (CALM1) or Endothelial NO synthase traffic inducer (NOSTRIN) [40]. This dysregulation may have an important hemodynamic impact on the coronary circulation, explaining why this polymorphism is associated with ACS clinical presentation.

Our results shed light on the idea that IHD is not the result of a single pathological process but the result of multiple districts’ involvement, justified by the presence of a complex network among the microcirculation and greater caliber arteries. Atherosclerotic disease, which mainly involves the epicardial arteries, may compromise vasal tone regulation, leading to CMD. On the contrary, CMD through hampered vasal tone dynamism and blood slowdown promotes atherosclerotic plaque formation and progression, in larger coronary arteries. In this context, endothelial dysfunction may represent the lowest common denominator responsible for the pathophysiological processes leading to IHD.

Actually, there have been few studies demonstrating that an eNOS SNP predicts myocardial ischemia susceptibility, through endothelial dysfunction, and its clinical presentation. In our opinion, the importance of identifying genetic predictors of myocardial ischemia, able to be associated with clinical presentation, is crucial for the identification of patients at high CV risk and for their prognostic stratification and correct management. In this context, our preliminary results may be helpful for genetic-based prevention strategies against myocardial ischemia, in the future. Moreover, these results can lead the way to new therapeutic targets against IHD. Our results encourage further studies to investigate the expression and function of SNPs for eNOS, as well as their interaction with CV risk factors in IHD susceptibility.

This study has several limitations. First, it was a single-center study, and subjects recruited had the same Caucasian ethnicity. The power calculation was performed in advance assuming the population at risk and allele frequencies in the population because of the scant presence of pre-existing data. There is a lack of data regarding the functional impact of this SNP on eNOS expression and function. The present study analyzed only a single SNP of the *NOS3* gene. We did not investigate other SNPs or other proteins involved in endothelial function, at the coronary level.

## 5. Conclusions

The results of this study highlight the role of the eNOS rs1799983 SNP as an independent genetic predictor of IHD and ACS clinical presentation, beyond underlying pathophysiological mechanisms of myocardial ischemia and CV risk factors. These data suggest that genetically determined endothelial dysfunction may be the *primum movens* for IHD determination, also in ACS patients.

In clinical practice, these results may be useful to predict IHD development and its acute manifestation occurrence. This may allow the early identification of patients at high risk of developing IHD and more prone to develop ACS, promoting a genetic-based prevention strategy against myocardial ischemia. Finally, rs1799983 SNP of eNOS may become a new therapeutic target, as part of a genetic-based therapeutic management.

## Figures and Tables

**Table 1:** Baseline features of the overall population, and differences between the ischemic groups (G1 and G2) and control group (G3).

	Overall Population (N = 362)	G1/G2 (N = 272)	G3 (N = 90)	*p*-Value
age (years)	64 ± 13	66 ± 12	59 ± 14	<0.001
male gender	245	199	46	<0.001
BMI (kg/m^2^)	25.9 ± 3.4	26.0 ± 2.9	25.5 ± 4.7	0.33
arterial hypertension	242	201	41	<0.001
diabetes mellitus	87	74	13	0.01
dyslipidemia	188	150	38	0.03
smoking habit	142	114	28	0.07
familial history of CV disease	134	113	21	0.001
AF	44	35	9	0.47
COPD	31	25	6	0.46
CKD	15	12	3	0.77
LVEF (%)	49 ± 11	48 ± 10	53 ± 10	<0.001
rs1799983_GG	158	115	43	0.36
rs1799983_GT	59	51	8	0.03
rs1799983_TT	145	106	39	0.46

BMI: body mass index; CV: cardiovascular; AF: atrial fibrillation; COPD: chronic obstructive pulmonary disease; CKD: chronic kidney disease; LVEF: left ventricular ejection fraction; rs1799983: polymorphism for the gene nitric oxide synthase 3.

**Table 2:** Baseline features of the ischemic population, and differences between CAD (G1) and CMD (G2) patients.

	IHD (N = 272)	G1 (N = 218)	G2 (N = 54)	*p*-Value
age (years)	66 ± 12	67 ± 12	62 ± 13	0.001
male gender	199	175	24	<0.001
BMI (kg/m^2^)	26.0 ± 2.9	26.0 ± 2.9	26.1± 2.9	0.77
arterial hypertension	201	152	49	0.001
diabetes mellitus	74	67	7	0.01
dyslipidemia	150	127	23	0.04
smoking habit	114	100	14	0.01
familial history of CV disease	113	96	17	0.12
AF	35	29	6	0.66
COPD	25	21	4	0.46
CKD	12	12	0	0.13
LVEF (%)	48 ± 10	46 ± 11	54 ± 7	<0.001
rs1799983_GG	115	92	23	1
rs1799983_GT	51	39	12	0.47
rs1799983_TT	106	87	19	<0.001

IHD: ischemic heart disease; BMI: body mass index; CV: cardiovascular; AF: atrial fibrillation; COPD: chronic obstructive pulmonary disease; CKD: chronic kidney disease; LVEF: left ventricular ejection fraction; rs1799983: polymorphism for the gene nitric oxide synthase 3.

**Table 3:** Predictors of ischemic heart disease in univariate and multivariate analyses. Beyond the role of traditional cardiovascular risk factors, the allelic variant GT of the nitric oxide synthase 3 gene single-nucleotide polymorphism (SNP) rs1799983 is an independent predictor of ischemic heart disease.

	Univariate			Multivariate		
	OR	95% CI	*p*-Value	OR	95% CI	*p*-Value
age (years)	1.045	[1.025–1.066]	<0.0001	1.042	[1.019–1.066]	<0.001
male gender	2.608	[1.593–4.268]	<0.0001	3.373	[1.873–6.074]	<0.001
arterial hypertension	3.383	[2.062–5.552]	<0.0001	4.713	[2.609–8.516]	<0.001
diabetes mellitus	2.214	[1.161–4.221]	0.02	1.370	[0.645–2.912]	0.41
dyslipidemia	1.682	[1.039–2.724]	0.03	1.261	[0.706–2.251]	0.43
smoking habit	1.598	[0.962–2.653]	0.07			
familial history of CV disease	2.335	[1.354–4.026]	0.002	2.228	[1.208–4.110]	0.01
AF	1.329	[0.612–2.884]	0.47			
COPD	1.417	[0.562–3.573]	0.46			
LVEF	0.941	[0.914–0.970]	<0.0001	0.956	[0.926–0.987]	0.006
rs1799983_GG	0.801	[0.496–1.292]	0.36			
rs1799983_GT	2.365	[1.076–5.197]	0.03	3.872	[1.555–9.637]	0.004
rs1799983_TT	0.835	[0.515–1.353]	0.46			

CV: cardiovascular disease; AF: atrial fibrillation; COPD: chronic pulmonary obstructive disease; LVEF: left ventricular ejection fraction; NOS3: nitric oxide synthase 3; OR: odds ratio; CI: confidence interval; rs1799983: polymorphism for the gene nitric oxide synthase 3.

**Table 4:** Predictive factors of acute coronary syndrome presentation in univariate and multivariate analyses. Beyond the role of traditional cardiovascular risk factors, the allelic variant GT of the nitric oxide synthase 3 gene single-nucleotide polymorphism (SNP) rs1799983 is an independent predictor of acute coronary syndrome presentation.

	Univariate			Multivariate		
	OR	95% CI	*p*-Value	OR	95% CI	*p*-Value
age (years)	1.024	[1.007–1.041]	0.005	1.016	[0.997–1.036]	0.10
male gender	1.492	[0.951–2.341]	0.08	1.319	[0.793–2.194]	0.29
arterial hypertension	1.590	[1.015–2.491]	0.04	1.917	[1.147–3.205]	0.01
diabetes mellitus	1.585	[0.976–2.572]	0.06	1.124	[0.649–1.948]	0.68
dyslipidemia	1.364	[0.899–2.070]	0.14			
smoking habit	1.778	[1.160–2.724]	0.008	1.571	[0.971–2.540]	0.07
familial history of CV disease	1.940	[1.259–2.989]	0.003	1.915	[1.185–3.094]	0.008
AF	1.176	[0.625–2.214]	0.62			
LVEF	0.937	[0.916–0.959]	<0.001	0.937	[0.915–0.961]	<0.001
rs1799983_GG	0.699	[0.458–1.064]	0.09	0.947	[0.571–1.571]	0.83
rs1799983_GT	2.259	[1.276–4.000]	0.005	2.821	[1.424–5.589]	0.003
rs1799983_TT	0.907	[0.594–1.386]	0.65			

CV: cardiovascular; AF: atrial fibrillation; LVEF: left ventricular ejection fraction; NOS3: nitric oxide synthase 3; OR: odds ratio; CI: confidence interval; rs1799983: polymorphism for the gene nitric oxide synthase 3.
